# Investigation of Morphometric Characteristics of the Mesencephalon in a Healthy Turkish Population: An MRI-Based Morphometric Study

**DOI:** 10.7759/cureus.48708

**Published:** 2023-11-12

**Authors:** Mehmet Demir, Selim Cinaroglu, Faruk G Ceranoğlu, Fatih Çiçek, Turan Koç

**Affiliations:** 1 Department of Anatomy, Kahramanmaraş Sütçü İmam University, Kahramanmaraş, TUR; 2 Department of Anatomy, Niğde Ömer Halisdemir University, Niğde, TUR

**Keywords:** pontomesencephalic angle, turkish population, magnetic resonance imaging, mesencephalon morphometry, age and sex differences

## Abstract

Introduction: Due to the important functions of the mesencephalon, knowledge of its morphometric characteristics in a healthy population is important for any pathological diagnosis. The aim of this study was to determine the specific morphometric values of the mesencephalon in a healthy Turkish population.

Methods: Magnetic resonance (MR) images of 184 subjects (98 females, 86 males) with a mean age of 47.33 years (range 18 to 85 years) were included in the study. Then, parameter measurements were performed on 1.5 T MR images, and MicroDicom Digital Imaging and Communications in Medicine (DICOM) viewer 2022.1 (MicroDicom Ltd., Sofia, Bulgaria) software program was used for the measurements.

Results: The mean sagittal diameter of the right cerebral peduncle was 17.17±2.03 mm, the mean cross-sectional area of the right cerebral peduncle was 171.75±32.81 mm^2^, the mean transverse diameter of the left cerebral peduncle was 16.60±2.32 mm, sagittal diameter of tectum and tegmentum 17.01±1.57 mm, the cross-sectional area of tectum and tegmentum 223.40±27.37 mm^2^ and pontomesencephalic angle 52.03°±10.61°, while in males these values were 18.26±2.38 mm, 182.61±38.57 mm^2^, 17.39±2.57 mm, 17.76±1.90 mm, 237.20±35.94 mm^2^ and 56.77°±9.78°, respectively. Except for the mamillopontine distance, there was a statistically significant difference between genders in the other parameters (p<0.05).

Conclusion: In conclusion, the findings related to the mesencephalon obtained in this study are presented for the first time in a healthy Turkish population. Especially, the cerebral peduncle cross-sectional area, tectum and tegmentum cross-sectional area, and cerebral peduncle transverse diameter can be evaluated clinically. We believe that knowledge of these values will guide specialists and radiologists in the diagnosis of any pathologic condition. Furthermore, the pontomesencephalic angle and mamillopontine distance have been identified as potentially useful landmarks in the diagnosis of intracranial hypotension and hydrocephalus. In particular, these angles can be measured in patient groups and may be a potential landmark in making an alternative diagnosis.

## Introduction

The brain stem consists of three parts, the medulla oblongata, pons, and mesencephalon. This structure connects the brain, cerebellum, and spinal cord [1-3]. Moreover, it plays a very important role in regulating motor, sensory, sympathetic, and parasympathetic effects, being the center of vital reflexes and being the exit point of cranial nerves [4]. The most rostral part of the brainstem, located between the pons and diencephalon, is the mesencephalon. The mesencephalon is divided into two parts, the cerebral peduncle and tectum mesencephalicum. The nuclei of cranial nerves III (oculomotor nerve) and IV (trochlear nerve) and part of the nucleus of cranial nerve V (trigeminal nerve) are located here. This part of the brain is the reflex center for both the eye and vision. It is also responsible for dopamine balance with the substantia nigra and voluntary movements with the red nucleus [1-3].

Normal brain development, maturation, and the effects of aging are well documented in the literature. Since the advent of magnetic resonance imaging (MRI), it has been possible to describe the structural anatomy and morphology of the brain with great accuracy [5]. A number of diseases, as well as the aging process, are known to be associated with a reduction in the size or atrophy of various structures of the brain. Diagnosis of such diseases often utilizes measurements from MR images, which are routinely and easily performed [4-7]. Measurements of various structures of the brain, especially those known to be associated with atrophy of the brainstem, play a critical role in the diagnosis of neurodegenerative pathologies. Pontomesencephalic angle (PMA) and mamillopontine distance (MPD) have been found useful in the diagnosis of intracranial hypotension. It has also been reported that MPD is a useful aid in the diagnosis of hydrocephalus [8-12].

In the literature review, it was noteworthy that there were not enough morphometric studies on the brainstem. In particular, no specific morphometric study on the mesencephalon in the Turkish population was found in the literature. For the reasons mentioned above, we believe that knowing the morphometric properties of the mesencephalon in healthy individuals will make an important contribution to radiologists and specialist physicians in the diagnosis of any pathology.

The aim of this study was to reveal the morphometric properties of the mesencephalon in a healthy Turkish population by using brain MR images and classifying them according to age and gender.

## Materials and methods

This study was approved by the Non-Interventional Ethics Committee of Niğde Ömer Halisdemir University (Ethic Approval code: 2023/09-05) and was conducted in accordance with the principles of the Declaration of Helsinki. A total of 184 people (98 female, 86 male) with MR images between December 2020 and November 2022 were included in the study. The mean age of the participants was 47.33 years (range 18 to 85 years). Patients between the ages of 18-85 who presented to the hospital with headache and underwent brain MRI without any pathology were included in the study, while those who had previously undergone intracranial surgery, had any tumor, etc. lesions/pathologies in the brain and brainstem were excluded from the study. The participants were divided into 6 groups according to their ages: 18-35 (Group 1), 36-45 (Group 2), 46-55 (Group 3), 56-65 (Group 4), 66-75 (Group 5) and 76-85 (Group 6).

**Figure 1:**
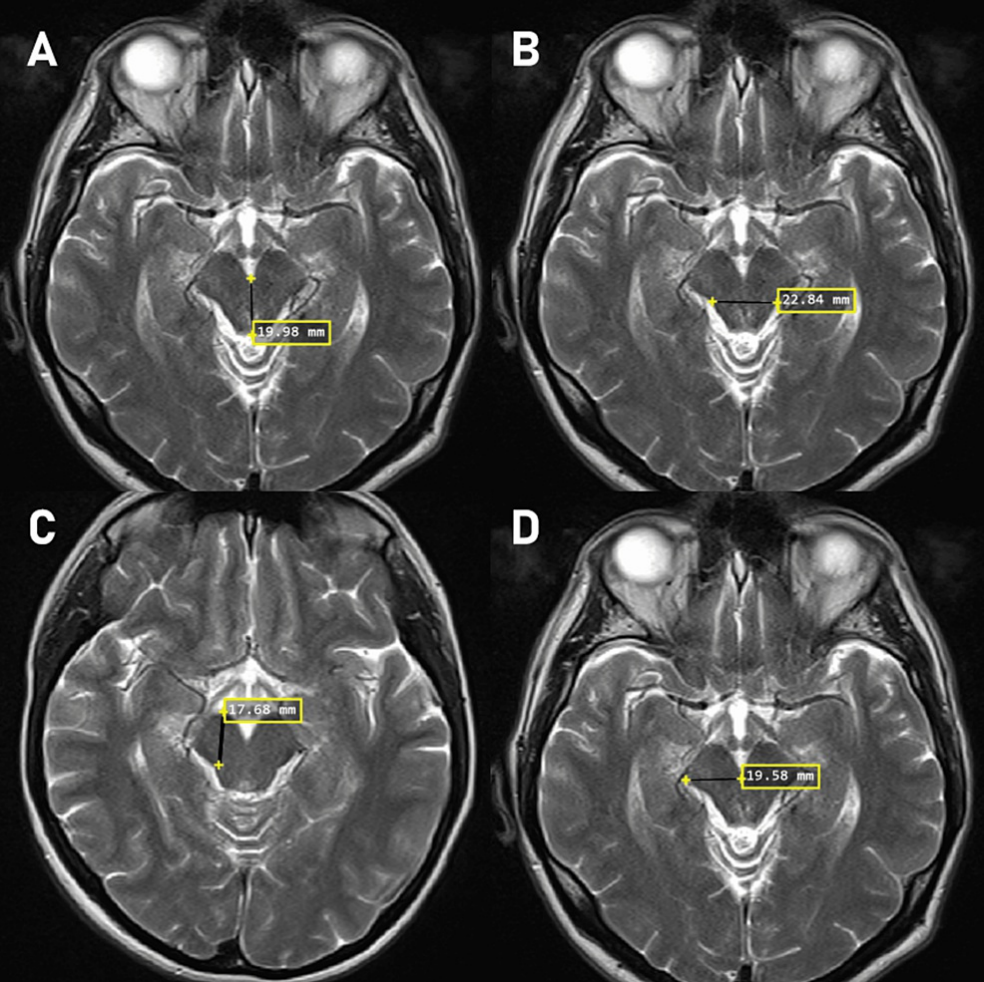
Mesencephalon morphometric measurements A: Tectum and tegmentum sagittal diameter, B: Tectum and tegmentum transverse diameter, C: Pedunculus cerebri sagittal diameter, D: Pedunculus cerebri transverse diameter

**Figure 2:**
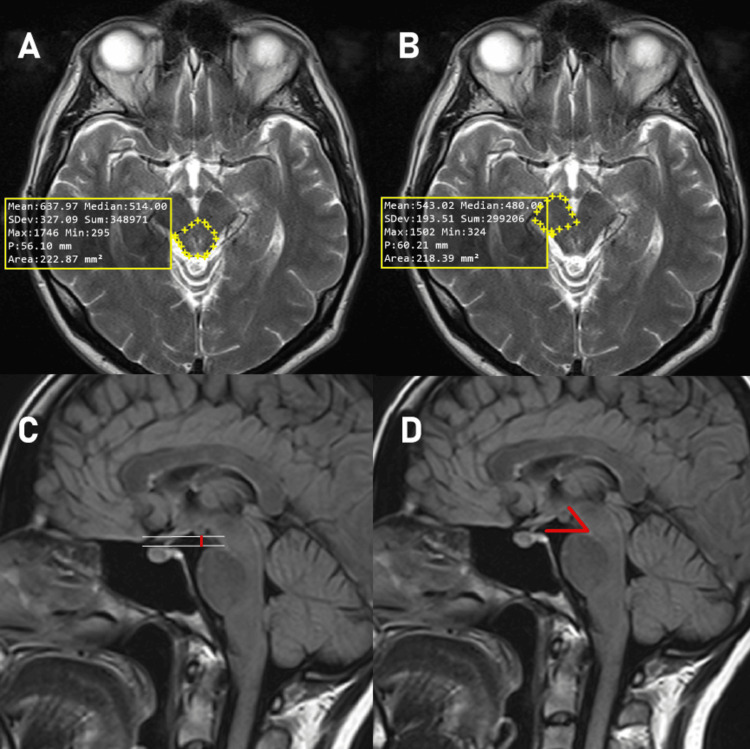
Mesencephalon morphometric measurements A: Tectum and tegmentum cross-sectional area, B: Pedunculus cerebri cross-sectional area, C: Mamillopontine distance, D: Pontomesencephalic angle

Parameters and reference points measured in the study are shown in Figure 1A-1D and Figure 2A-2D.

The sagittal diameter of tegmentum and tectum (SDTT) (mm), that is the distance between the anterior and posterior-most point of the tegmentum and tectum at the level of the chiasma opticum on T2 sequence and transverse plane is shown in Figure 1A. The tegmentum and tectum transverse diameter (TDTT) (mm), that is the distance from the most medial to the most lateral point of the tegmentum and tectum at the level of the chiasma opticum on T2 sequence and transverse plane is shown in Figure 1B. The pedunculus cerebri sagittal diameter (SDPC) (mm), that is the distance from the most anterior to the most posterior point of the pedunculus cerebri at the level of the chiasma opticum on T2 sequence and transverse plane is shown in Figure 1C. The pedunculus cerebri transverse diameter (TDPC) (mm), that is the distance from the most medial to the most lateral point of the pedunculus cerebri at the level of the chiasma opticum on T2 sequence and transverse plane is shown in Figure 1D.

The cross-sectional area of tegmentum and tectum (CSATT) (mm^2^), that is the total surface area of the tegmentum and tectum at the level of the chiasma opticum on T2 sequence and transverse plane is shown in Figure 2A. The cross-sectional area of the pedunculus cerebri (CSAPC) (mm^2^), that is the surface area of the pedunculus cerebri at the level of the chiasma opticum on T2 sequence and transverse plane is shown in Figure 2B. The mamillopontine distance (MPD) (mm), defined as the distance between a transverse line drawn from the lowest point of the corpus mamillare and a transverse line drawn from the highest point of the pons on T2 sequence and sagittal plane [11] is shown in Figure 2C. The pontomesencephalic angle (PMA) (°), defined as the angle between the line drawn along the anterior edge of the mesencephalon and the anterior superior edge of the pons in T2 sequence and sagittal plane [11] is shown in Figure 2D.

All parameter measurements were performed on 1.5 T MR images, and MicroDicom Digital Imaging and Communications in Medicine (DICOM) viewer 2022.1 (MicroDicom Ltd., Sofia, Bulgaria) was used for the measurements.

Statistical analysis

Statistical Package for Social Sciences (SPSS), version 23.0 (IBM Corp. Armonk, NY) software program was used in the statistical analysis of the study. Kolmogorov-Smirnov test was used to determine whether the data obtained were normally distributed. Then, it was determined that the data were normally distributed. Therefore, an independent sample t-test was used for the comparison of the parameters between genders, and a one-way ANOVA test was used for the comparison between age groups.

## Results

The mean and standard deviation values of the measurements related to the mesencephalon in males and females and whether there was a significant difference between the two genders are presented in Table 1. Accordingly, it was found to be statistically significant (p˂0.05) that males had higher mean values than females in all other brainstem measurements except MPD (Table 1). The mean value of MPD was also found to be greater in males than females, but not statistically significant (p= 0.709) (Table 1).

**Table 1:** Distribution of mesencephalon-related parameters according to gender SDRPC: Right side pedunculus cerebri sagittal diameter; TDRPC: Right side pedunculus cerebri transverse diameter; CSARPC: Right side pedunculus cerebri cross-sectional area; SDLPC: Left side pedunculus cerebri sagittal diameter; TDLPC: Left side pedunculus cerebri transverse diameter; CSALPC: Left side pedunculus cerebri cross-sectional area; SDTT: Tectum+tegmentum sagittal diameter; TDTT: Tectum+tegmentum transverse diameter; CSATT: Tectum+tegmentum cross-sectional area; MPD: Mamillopontine distance; PMA: Pontomesencephalic angle

Parameters	Female (N=98) (Mean±SD)	Male (N=86) (Mean±SD)	p
SDRPC	17.17 ±2.03	18.26±2.38	0.001
TDRPC	16.62±2.23	17.33±2.50	0.043
CSARPC	171.75±32.81	182.61±38.57	0.041
SDLPC	16.45±1.78	18.13±2.24	<0.001
TDLPC	16.60±2.32	17.39±2.57	0.029
CSALPC	164.53±32.12	183.02±36.02	<0.001
SDTT	17.01±1.57	17.76±1.90	0.004
TDTT	21.52±1.84	22.93±2.47	<0.001
CSATT	223.40±27.37	237.20±35.94	0.004
MPD	6.39±1.01	6.45±0.99	0.709
PMA	52.03±10.61	56.77±9.78	0.002

The comparison of PMA/MPD, tectum+tegmentum transverse diameter/tectum+tegmentum sagittal diameter (TDTT/SDTT), right side pedunculus cerebri cross-sectional area/left side pedunculus cerebri cross-sectional area (CSARPC/CSALPC) ratios between genders are shown in Table 2. The mean values of PMA/MPD and TDTT/SDTT were higher in males than females, and the mean value of CSARPC/CSALPC was higher in females than males. However, there was no statistically significant difference in all rates (p>0.05) (Table 2).

**Table 2:** Distribution of the rates of mesencephalon-related measurements according to gender PMA/MPD: Pontomesencephalic angle/mamillopontine distance; TDTT/SDTT: Tectum+tegmentum transverse diameter/tectum+tegmentum sagittal diameter; CSARPC/CSALPC: Right side pedunculus cerebri cross-sectional area/left side pedunculus cerebri cross-sectional area

Parameters	Female (N=98) (Mean±SD)	Male (N=86) (Mean±SD)	p
PMA/MPD	8.38±2.40	8.91±1.61	0.078
TDTT/SDTT	1.27±0.16	1.29±0.13	0.336
CSARPC/CSALPC	1.05±0.18	1.00±0.18	0.063

Table 3 shows the mean, standard deviation, and statistical significance values of measurements related to the mesencephalon in all age groups. It was found to be statistically significant that the sagittal diameter of the right side pedunculus cerebri (SDRPC) decreased with increasing age in both sexes (p=0.001) (Table 3). In addition, there was a statistically significant difference in left side pedunculus cerebri sagittal diameter (SDLPC), left side pedunculus cerebri transverse diameter (TDLPC), CSALPC, MPD, and PMA parameter values between both sexes and age groups (p˂0.05) (Table 3).

**Table 3:** Distribution of measurements related to mesencephalon according to age groups SDRPC: Right side pedunculus cerebri sagittal diameter; TDRPC: Right side pedunculus cerebri transverse diameter; CSARPC: Right side pedunculus cerebri cross-sectional area; SDLPC: Left side pedunculus cerebri sagittal diameter; TDLPC: Left side pedunculus cerebri transverse diameter; CSALPC: Left side pedunculus cerebri cross-sectional area; SDTT: Tectum+tegmentum sagittal diameter; TDTT: Tectum+tegmentum transverse diameter; CSATT: Tectum+tegmentum cross-sectional area; MPD: Mamillopontine distance; PMA: Pontomesencephalic angle

Parameters	Group 1 (N=46) (Mean±SD)	Group 2 (N=46) (Mean±SD)	Group 3 (N=34) (Mean±SD)	Group 4 (N=28) (Mean±SD)	Group 5 (N=22) (Mean±SD)	Group 6 (N=22) (Mean±SD)	p
SDRPC	18.71±1.66	18.09±3.13	17.18±1.65	16.95±1.84	16.81±1.96	16.46±0.47	0.001
TDRPC	16.83±2.33	17.06±2.42	16.83±2.26	16.63±2.62	17.76±2.67	16.48±0.97	0.601
CSARPC	185.43±34.39	181.03±38.70	174.81±28.08	168.35±34.80	171.96±46.83	154.75±3.88	0.136
SDLPC	17.70±2.34	18.00±2.49	17.43±1.71	15.90±1.62	16.53±1.50	15.95±1.08	<0.001
TDLPC	17.10±2.43	16.66±1.93	17.21±2.27	16.25±2.50	18.27±3.25	15.96±2.70	0.048
CSALPC	175.76±37.32	183.34±41.22	177.40±27.74	156.75±24.97	169.10±34.62	150.56±15.97	0.012
SDTT	17.47±1.85	17.67±1.85	17.50±1.50	16.79±1.70	17.09±2.07	17.10±0.89	0.373
TDTT	21.57±2.42	22.34±2.33	21.89±1.81	22.51±1.83	23.02±2.86	22.50±1.67	0.162
CSATT	233.30±34.14	241.06±31.75	228.82±28.75	233.41±34.50	218.85±32.24	225.18±12.99	0.060
MPD	6.43±1.00	6.37±1.04	6.13±1.04	6.97±0.99	6.37±0.80	6.12±0.31	0.031
PMA	55.14±9.57	55.37±9.27	51.98±9.35	50.11±12.89	54.58±9.51	65.83±11.90	0.004

Table 4 shows the mean, standard deviation, and statistical significance values of the ratios of mesencephalon-related measurements in all age groups. While PMA/MPD and TDTT/SDTT ratios were statistically significant between age groups in both sexes (p˂0.05), no significant difference was found between age groups of both sexes in the CSARPC/CSALPC ratio (p>0.05) (Table 4).

**Table 4:** Examination of the distribution of the rates of mesencephalon-related measurements according to age groups PMA/MPD: Pontomesencephalic angle/mamillopontine distance; TDTT/SDTT: Tectum+tegmentum transverse diameter/tectum+tegmentum sagittal diameter; CSARPC/CSALPC: Right side pedunculus cerebri cross-sectional area/left side pedunculus cerebri cross-sectional area

Parameters	Group 1 (N=46) (Mean±SD)	Group 2 (N=46) (Mean±SD)	Group 3 (N=34) (Mean±SD)	Group 4 (N=28) (Mean±SD)	Group 5 (N=22) (Mean±SD)	Group 6 (N=22) (Mean±SD)	p
PMA/MPD	8.66±1.41	8.82±1.74	8.83±2.69	7.36±2.38	8.66±1.72	10.78±2.12	0.001
TDTT/SDTT	1.24±0.15	1.27±0.14	1.25±0.12	1.35±0.17	1.35±0.14	1.31±0.08	0.009
CSARPC/CSALPC	1.07±0.19	1.00±0.19	0.98±0.08	1.08±0.21	1.02±0.21	1.03±0.08	0.160

Table 5 shows the mean, standard deviation, and statistical significance values of measurements related to mesencephalon in all age groups in males. When Table 5 is evaluated, it is seen that there is a statistically significant difference between age groups in SDRPC, SDLPC, TDLPC, and MPD parameters in male gender (p˂0.05).

**Table 5:** Distribution of mesencephalon-related diameters and ratios of mesencephalon-related measurements according to age groups in males (N=86) SDRPC: Right side pedunculus cerebri sagittal diameter; TDRPC: Right side pedunculus cerebri transverse diameter; CSARPC: Right side pedunculus cerebri cross-sectional area; SDLPC: Left side pedunculus cerebri sagittal diameter; TDLPC: Left side pedunculus cerebri transverse diameter; CSALPC: Left side pedunculus cerebri cross-sectional area; SDTT: Tectum+tegmentum sagittal diameter; TDTT: Tectum+tegmentum transverse diameter; CSATT: Tectum+tegmentum cross-sectional area; MPD: Mamillopontine distance; PMA: Pontomesencephalic angle

Parameters	Group 1 (N=20) (Mean±SD)	Group 2 (N=28) (Mean±SD)	Group 3 (N=10) (Mean±SD)	Group 4 (N=10) (Mean±SD)	Group 5 (N=16) (Mean±SD)	Group 6 (N=2) (Mean±SD)	p
SDRPC	18.50±2.32	19.19±2.87	17.99±1.17	17.92±1.14	16.91±2.20	16.52±0.00	0.047
TDRPC	16.83±2.36	17.67±2.70	17.48±2.58	17.95±2.03	17.12±2.73	15.40±0.00	0.667
CSARPC	183.45±41.63	187.09±34.43	195.32±21.82	180.73±41.33	170.72±48.69	152.50±0.00	0.526
SDLPC	18.46±2.57	18.81±2.55	18.97±0.69	17.19±1.26	16.85±1.63	15.96±0.00	0.017
TDLPC	17.47±2.67	17.13±1.90	17.23±2.00	18.06±1.41	18.15±3.60	11.76±0.00	0.030
CSALPC	182.02±40.37	190.80±42.19	190.76±11.56	175.82±16.91	175.72±36.59	140.06±0.00	0.343
SDTT	17.82±2.11	18.15±2.07	17.91±0.68	17.59±0.49	17.21±2.40	16.24±0.00	0.567
TDTT	22.33±2.91	23.16±2.15	23.54±0.43	23.71±1.99	22.80±3.24	19.88±0.00	0.298
CSATT	234.95±42.39	250.14±32.61	235.61±28.59	244.86±28.31	217.02±35.53	209.47±0.00	0.059
MPD	6.90±1.13	6.43±1.07	5.63±0.51	6.80±0.71	6.20±0.75	6.55±0.00	0.018
PMA	58.82±9.60	56.79±9.25	53.29±5.62	53.59±14.84	56.67±8.73	70.17±0.00	0.225
PMA/MPD	8.59±1.13	8.93±1.44	9.57±1.73	8.01±2.44	9.21±1.59	10.71±0.00	0.122
TDTT/SDTT	1.26±0.16	1.28±0.14	1.31±0.05	1.35±0.10	1.33±0.14	1.22±0.00	0.481
CSARPC/CSALPC	1.00±0.85	1.01±0.25	1.02±0.06	1.03±0.25	0.96±0.14	1.09±0.00	0.916

Table 6 describes the values of measurements related to the mesencephalon in females in all age groups. According to these findings, there is a statistically significant difference between age groups in SDRPC, TDRPC, SDLPC, TDLPC, TDLPC, TDTT, MPD, PMA, PMA/MPD, TDTT/SDTT, CSARPC/CSALPC parameters in female gender (p˂0.05) (Table 6).

**Table 6:** Distribution of mesencephalon-related diameters and ratios of mesencephalon-related measurements according to age groups in females (N=98) SDRPC: Right side pedunculus cerebri sagittal diameter; TDRPC: Right side pedunculus cerebri transverse diameter; CSARPC: Right side pedunculus cerebri cross-sectional area; SDLPC: Left side pedunculus cerebri sagittal diameter; TDLPC: Left side pedunculus cerebri transverse diameter; CSALPC: Left side pedunculus cerebri cross-sectional area; SDTT: Tectum+tegmentum sagittal diameter; TDTT: Tectum+tegmentum transverse diameter; CSATT: Tectum+tegmentum cross-sectional area; MPD: Mamillopontine distance; PMA: Pontomesencephalic angle

Parameters	Group 1 (N=26) (Mean±SD)	Group 2 (N=18) (Mean±SD)	Group 3 (N=24) (Mean±SD)	Group 4 (N=18) (Mean±SD)	Group 5 (N=6) (Mean±SD)	Group 6 (N=6) (Mean±SD)	p
SDRPC	18.87±0.91	16.38±2.78	16.84±1.73	16.42±1.96	16.54±1.21	16.44±0.55	<0.001
TDRPC	16.83±2.36	16.10±1.52	16.55±2.12	15.89±2.52	19.48±1.64	16.84±0.84	0.019
CSARPC	186.96±28.40	171.60±43.90	166.27±26.19	161.47±29.65	175.28±45.60	155.50±4.29	0.084
SDLPC	17.12±2.01	16.74±1.81	16.79±1.61	15.18±1.34	15.66±0.52	15.95±1.28	0.005
TDLPC	16.82±2.23	15.93±1.78	17.20±2.41	15.24±2.43	18.60±2.30	17.37±0.87	0.009
CSALPC	170.94±34.82	171.74±37.91	171.83±30.69	146.15±22.49	151.46±22.42	154.07±17.27	0.053
SDTT	17.20±1.63	16.92±1.12	17.33±1.71	16.34±1.97	16.76±0.80	17.38±0.85	0.409
TDTT	20.99±1.81	21.07±2.05	21.20±1.72	21.85±1.39	23.62±1.47	23.38±0.51	0.002
CSATT	214.34±23.24	226.93±25.16	225.98±28.94	227.06±36.69	223.74±23.19	230.42±10.23	0.547
MPD	6.06±0.72	6.28±1.00	6.34±1.13	7.06±1.12	6.81±0.85	5.98±0.19	0.020
PMA	52.32±8.70	53.17±9.13	51.43±10.58	48.17±11.68	49.01±10.01	64.38±13.72	0.041
PMA/MPD	8.72±1.60	8.66±2.17	8.51±2.98	7.01±2.33	7.19±1.12	10.80±2.50	0.011
TDTT/SDTT	1.23±0.14	1.25±0.15	1.23±0.14	1.35±0.20	1.41±0.15	1.34±0.06	0.015
CSARPC/CSALPC	1.12±0.24	0.99±0.04	0.97±0.08	1.11±0.18	1.16±0.31	1.01±0.91	0.007

## Discussion

The brain stem, which consists of three parts, the medulla oblongata, pons, and mesencephalon, has a small area in the central nervous system. However, it is of vital importance as it has functions such as respiratory and circulatory centers, swallowing and vocalization, vision, hearing reflex, and pupillary reflex [13-15]. It is also an important part of the nervous system where the pathways connecting the spinal cord to the cortex and the cortex to the spinal cord pass and neurons change [16]. The mesencephalon is the shortest and uppermost part of the brain stem. It extends between the aqueduct of midbrain tectum and tegmentum sections connecting the 3rd ventricle to the 4th ventricle in the CSF circulation. The nucleus ruber and substantia nigra, two important nuclei responsible for voluntary movements, the nuclei of the 3rd (n. oculomotorius), 4th (n. trochlearis) cranial nerves and a nucleus of the 5th (n. trigeminus) cranial nerve are located here [16-18].

In our study, the detailed morphometric values of the mesencephalon were examined in a healthy Turkish population, which is the first of its kind in the literature among certain age groups in males and females. Table 1 shows that the measurements of the mesencephalon, except for MPD, were statistically significantly higher in males than females (p ˂0.05).

Kariev et al. (2019) reported that MPD is an alternative measurement with high specificity in the diagnosis of obstructive hydrocephalus [10]. In another study with 43 patients and 30 control groups, the mean MPD value of the patient group was found to be 3.4 mm and 9.8 mm in the control group [5]. In our study conducted in a healthy Turkish population, it was determined that the mean MPD value of males was 6.45 mm, and that of females was 6.39 mm. However, there was no statistically significant difference between genders (p>0.05). In addition, according to the results of our study, it is shown in Table 3 that there is a statistically significant difference in MPD value according to age groups (p ˂0.05). The highest value was 6.97 mm in the 56-65 age group (Group 4) and the lowest value was 6.12 mm in the 76-85 age group (Group 6). In the study conducted by Debnath et al. (2022) in a healthy Indian population, it was found that the mean MPD value of males was 8.60 mm and 7.92 mm in females and there was a statistically significant difference between the sexes (p˂0.05) [5]. The results of this study conducted in an Indian population are different from those of our study.

Shah et al. (2013) reported that MPD values of 5.5 mm and less and PMA values of 50° and less were specific values suggestive of intracranial hypotension [11]. In our study conducted in a healthy Turkish population, it was found to be statistically significant (p˂0.05) that the mean PMA value was 56.77° in males and 52.03° in females (Table 1). There was also a statistically significant difference between age groups in terms of PMA value. When the PMA value was analyzed in age groups in males and females, the highest age group was group 6 with 70.17° and the lowest age group was group 3 with 53.29° and there was no statistically significant difference in terms of age groups (p>0.05), while in females, the highest age group was group 6 with 64.38° and the lowest age group was group 4 with 48.17° and there was a statistically significant difference between age groups (p˂0.05) (Tables 5, 6). In addition, the PMA/PMD ratio was also analyzed in our study. This value was found to be 8.91 in males and 8.38 in females and no statistical difference was found between both sexes (Table 2). We think that knowing this ratio in healthy subjects can help evaluate pathologic conditions

Semnic et al. (2005) reported that the transverse diameter of the pedunculus cerebri was 93% reliable in the diagnosis of Wilson's disease [19]. They reported that the mean transverse diameter of the pedunculus cerebri was 11 mm in the patient group and 12.9 mm in the control group of healthy subjects [19]. In our study, the transverse diameter of the pedunculus cerebri was measured separately on the right and left sides. In females, the mean value of the transverse diameter of the pedunculus cerebri on the right side was 16.62 mm and the mean diameter of the pedunculus cerebri on the left side was 16.60 mm. In males, this value was 17.33 mm on the right side and 17.39 mm on the left side, and there was a statistically significant difference between males and females on both the right and left sides (p˂0.05). In addition, in our study, the transverse diameter of the pedunculus cerebri on the right side in males and females was examined separately between age groups and there was no statistically significant difference between the age groups, while there was a significant difference between the age groups on the left side (Table 5). In females, the statistical difference between the transverse diameter of the pedunculus cerebri on both the right and left sides was statistically significant between age groups (Table 6). In addition to these findings, the ratio of the transverse diameter of the right side pedunculus cerebri to the transverse diameter of the left side pedunculus cerebri was examined and it was found that this ratio was 1 in males and 1.05 in females. However, this ratio is not statistically significant (Table 2). We think that knowing this ratio in healthy subjects can be evaluated in the detection of pathologic conditions. In the literature, it has been reported that various structures of the brain undergo atrophy in some diseases as well as the aging process [4-7].

In our study, the cross-sectional areas of the mesencephalon at the level of chiasma opticum, which are not available in the literature, were revealed between age groups in male and female sex. The cross-sectional area of the pedunculus cerebri on the right side was highest in group 3 with 195.32 mm^2^ in males and lowest in group 6 with 152.50 mm^2^ in females. On the left side, the highest age group was group 2 with 190.80 mm^2^ and the lowest age group was group 6 with 140.06 mm^2^. In the tectum and tegmentum cross-sectional area, the age group with the highest value was group 2 with 250.14 mm^2^ and the age group with the lowest value was group 5 with 209.47 mm^2^. The age group with the highest cross-sectional area of the pedunculus cerebri on the right side was group 1 with 186.96 mm^2^ and the lowest was group 6 with 155.50 mm^2^. On the left side, the highest age group was group 3 with 171.83 mm^2^ and the lowest age group was group 4 with 146.15 mm^2^. In the tectum and tegmentum cross-sectional area, the highest value of 230.42 mm^2^ belonged to group 6 (76-85 years), while the lowest value of 214.34 mm^2^ belonged to group 1 (18-35 years). We believe that knowing these values in both sexes and age groups will be an important guide in revealing the presence of any pathology, whether symptomatic or not.

## Conclusions

In conclusion, this is the first study in which pontomesencephalic angle/mamillopontine distance, tectum and tegmentum transverse diameter/tectum and tegmentum sagittal diameter, right pedunculus cerebri cross-sectional area/left pedunculus cerebri cross-sectional area ratios, pedunculus cerebri cross-sectional area and tectum and tegmentum cross-sectional area values were revealed in healthy Turkish population. We are of the opinion that revealing the specific morphometric values of the mesencephalon in the healthy Turkish population in detail, both in male and female gender and between different age groups, will be a guide for specialists and radiologists in the differential diagnosis of any pathological condition and surgical applications.
